# “Fight-flight-or-freeze” – how *Yarrowia lipolytica* responds to stress at molecular level?

**DOI:** 10.1007/s00253-022-11934-x

**Published:** 2022-04-30

**Authors:** Ewelina Celińska

**Affiliations:** Department of Biotechnology and Food Microbiology, Poznan University of Life Sciences, Wojska Polskiego 48, 60-627 Poznan, Poland

**Keywords:** Stress response, Environmental stress, Metabolic burden, Heterologous protein, Recombinant protein secretion, Yeast

## Abstract

**Abstract:**

*Yarrowia lipolytica* is a popular yeast species employed in multiple biotechnological production processes. High resistance to extreme environmental conditions or metabolic burden triggered by synthetically forced over-synthesis of a target metabolite has its practical consequences. The proud status of an “industrial workhorse” that *Y. lipolytica* has gained is directly related to such a quality of this species. With the increasing amount of knowledge coming from detailed functional studies and comprehensive omics analyses, it is now possible to start painting the landscape of the molecular background behind stress response and adaptation in *Y. lipolytica*. This review summarizes the current state-of-art of a global effort in revealing how *Y. lipolytica* responds to both environmental threats and the intrinsic burden caused by the overproduction of recombinant secretory proteins at the molecular level. Detailed lists of genes, proteins, molecules, and biological processes deregulated upon exposure to external stress factors or affected by over-synthesis of heterologous proteins are provided. Specificities and universalities of *Y. lipolytica* cellular response to different extrinsic and intrinsic threats are highlighted.

**Key points:**

*• Y. lipolytica as an industrial workhorse is subjected to multiple stress factors*.

*• Cellular responses together with involved genes, proteins, and molecules are reviewed*.

*• Native stress response mechanisms are studied and inspire engineering strategies*.

## Introduction

*Yarrowia lipolytica*, which is commonly used as a protein over-production platform, is often claimed to be an extremophile yeast species (Bankar et al. 2009). It is frequently isolated from a range of challenging natural environments and shows resistance to extreme conditions simulated in the laboratory, such as ambient pH from 2.5 up to 9.5, high salinity/osmolality, elevated temperatures up to 38 ℃, or presence of toxic compounds (Andreishcheva et al. 1999; Walker et al. 2019; Madzak 2021; Qiu et al. 2021; Sekova et al. 2021). High resistance to severe environmental conditions has its practical consequences in biotechnological processes, especially in relation to the occurrence of unavoidable gradients of temperature, pH, oxygen, osmolality, and concentration of chemical compounds, of different nature, intensity, duration, and/or frequency. Environmental stress factors strongly impact the overall performance of the microbial producer cells, including their growth rate, metabolic activity, and production capacity. Aside from external threats, a yeast producer cell operating in a targeted biotechnological production process is subjected to intrinsic stress triggered by synthetically enforced flux toward a specific biological process. For example, excessive production of a recombinant (secretory) protein (r(s)-Prot) frequently leads to the accumulation of unfolded polypeptides and endogenous stress (Mattanovich et al. 2004; Matsumoto et al. 2005; Tyo et al. 2012; Hou et al. 2014), or even instability of the producer cells (Ogrydziak and Nicaud 2012).

It is well recognized that the manifestation of a “resistant”/”efficient over-producer” phenotype is rarely founded by the favorable operation of a single or several genes. Usually, numerous molecular events must be fine-tuned to develop such functionality. While identification of the key molecular players in a given process and characterization of mechanisms of their involvement provides new, exciting basic knowledge, it also bears significant implications to be further developed in the applied research field and industrial practice. Mechanisms of the endogenous or environmental stress response are well described for *Saccharomyces cerevisiae* (Gasch et al. 2000; Gasch and Werner-Washburne 2002; Gasch 2007), unrevealing the major molecular players in these processes (Craig et al. 1993; Verghese et al. 2012). Interestingly, recent studies conducted with the model yeast species demonstrated a high overlap between the response to environmental stress (heat shock) and endogenous stress triggered by over-synthesis of rs-Prot (unfolded protein response—UPR) (Hahn et al. 2004; Guyot et al. 2005; Verghese et al. 2012; Hou et al. 2013). It has been evidenced that regulome induced by the heat shock covers 3% of the *S. cerevisiae* genome, out of which over 25% is represented by proteins involved in translational-secretory machinery, which are also involved in the UPR (Hahn and Thiele 2004; Hahn et al. 2004). Interestingly, it has been demonstrated that induction of the heat shock response improves rs-Prots secretion in *S. cerevisiae* (Hou et al. 2013).

**Fig. 1:**
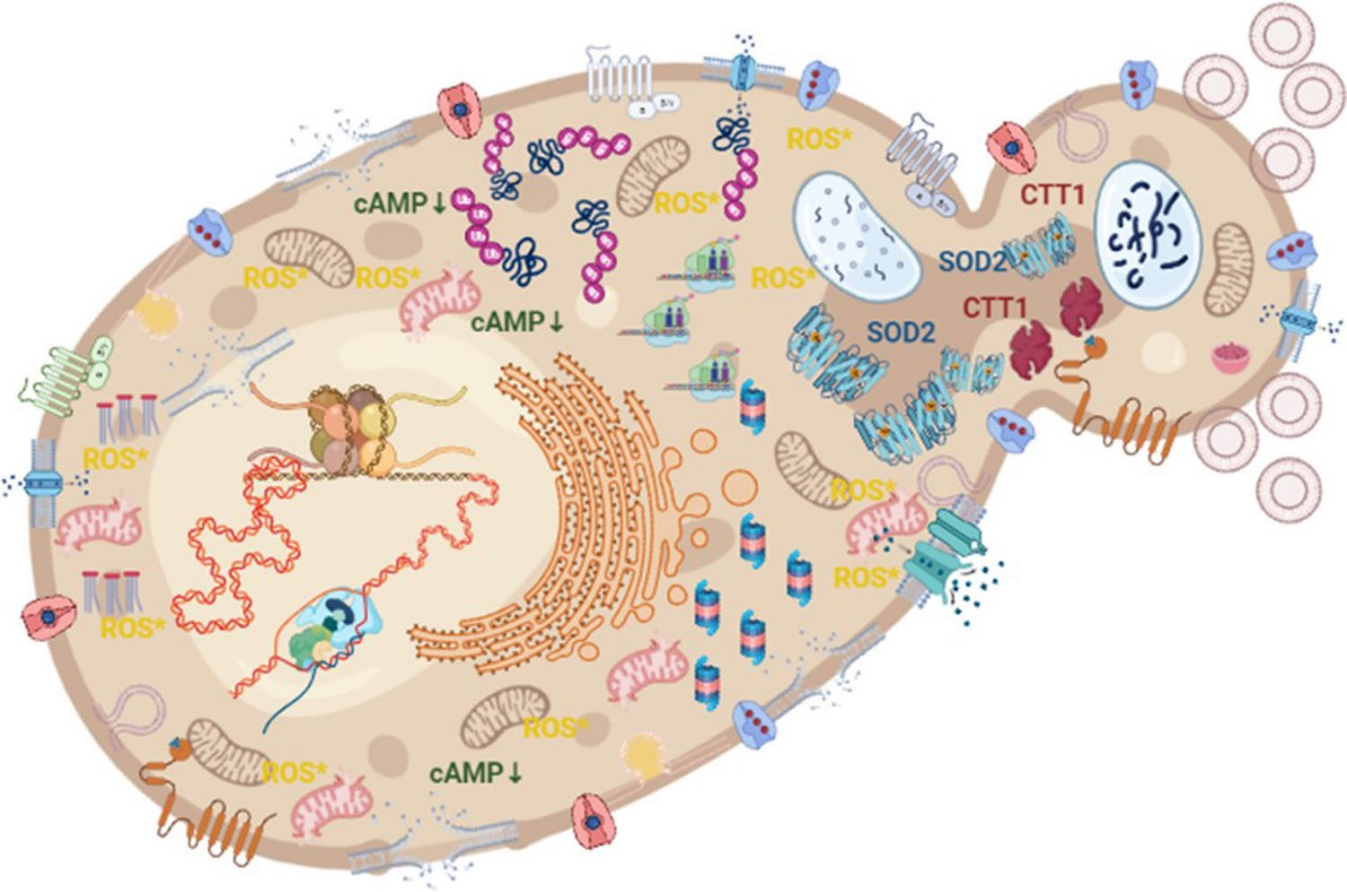
Schematic representation of processes involved in general stress-response in *Y. lipolytica*. Highlighted biological processes: structural and compositional changes to the cell membrane, loss of cell integrity; structural and compositional changes to cell wall – formation of protruding elements, enhanced formation of extracellular vesicles; cell membrane invagination hallmarking qualitative and quantitative modifications of channels and sensor/changes to cell size; enrichment of biological processes localized to cell surface hallmark possible dimorphic transition; oxidative degradation of lipids, proteins, and DNA; ubiquitination, proteasomal degradation, and vacuolar lysis of proteins; mitochondria over-loading and outburst of the oxidative stress response; chromatin structure modifications and changes to genes expression/protein abundance profiles; activation of membrane transporters and ion pumps

Nowadays, with the increasing scientific knowledge on *Y. lipolytica* coming from detailed molecular and functional studies, as well as global omics analyses, it seems possible to start painting the molecular landscape of stress response in this yeast species. This review summarizes the current state-of-art of a global effort in revealing how *Y. lipolytica* responds to both environmental threats and the intrinsic burden caused by the overproduction of rs-Prots at the molecular level. This article aims at answering the following questions: Which biological processes are affected? Which genes/proteins and molecules are used to combat unfavorable conditions and to adapt? Due to the known interconnection of environmental stress response and synthesis of rs-Prot, the two processes will be discussed hereafter as extrinsic and intrinsic stressors, respectively. All gene/protein names refer to a reference genome version YALI0, available at http://gryc.inra.fr/, and hence, only shortened numbers are provided. A simplified scheme of *Y. lipolytica*’s cellular response to various stress factors is illustrated in Fig. 1. Table 1 provides a summary of the main physiological and molecular phenomena awaken by specific stress factors.

**Table 1:** Summary of the main physiological and molecular phenomena awaken by specific stress factors in *Y. lipolytica* cells

Stress factor	Cellular response	References
Low-oxygen availability		
Different pO2 levels	Glucose concentration-dependent filamentation mediated via RAS-cAMP-PKA; in the presence of cAMP – no filamentation	(Ruiz-Herrera and Sentandreu 2002; Bellou et al. 2014; Timoumi et al. 2017a, 2018; Gorczyca et al. 2020; Lesage et al. 2021)
	Downregulation of lipid biosynthesis	
	Restorable growth rate limitation	
Acidity – pH		
pH 3.0	Higher energy requirement	(Madshus 1988; Cogo et al. 2020)
	Increased abundance and activity of plasma membrane H + -ATPase Pma1p	
pH 4.0/3.0	Intracellular proton extrusion across the mitochondrial inner membrane – the major mechanism contributing to the pH homeostasis	(Guo et al. 2016)
	Increased energy demand – upregulation of glycolysis -– Pgk1p, Gut2p, Eno2p, Tpi1p, Tdh3p, Fba1p, Pck1p	
	Key role of mitochondria (and increased energy demand): Upregulation of mitochondrial proteins – Por1p, Cit1p, Pda1p, Pdb1p, Mdh1p, Icl1p, Kgd2p, Acs2p Upregulation in mitochondrial electron transport chain and ATP synthesis – Nuamp, Nuemp, Nufmp, Qcr2p, Atp1-2–3-7p, Cdc48p, Afg1p	
	Onset of oxidative stress response: Increased synthesis of amino acids– Aat2p, Leu1p, Gcv2p, Sam2p, Met6p, Ilv5p, Ses1p, Gat1p, Shm1p Upregulation of molecular chaperones (Kar2p, Sse1p, Ssa4p) Upregulation of Sod2p	
	Enhanced synthesis of aKGA	
pH 9.0	Drop in intracellular sugar amounts by 25%	(Sekova et al. 2019)
	Changes in intracellular sugar composition – increase in MAN, no glucose, no TRE	
	Drop in storage lipids – lipid bodies and/or membrane lipids by ~ 30% and 36%	
	Increased level of saturated fatty acids (sFAs) in mitochondria	
	Upregulation of Sod2p activity	
	Reduced glutathione (GSH) concentration	
pH 9.0	Increased chaperoning capacity – rotamase, Hsps	(Sekova et al. 2021)
	Substantial changes in mitochondria activity – upregulation of malate dehydrogenase, VDAC porin, NADPH dehydrogenase, mitochondrial chaperones, and pore constituents Mitochondrial VDAC was deemed as one of the key proteins responding to the alkaline pH	
	Enhanced energy demand – upregulation of TPI1/Tpi1p and GAPDH	
pH 4.5/pH 7.0	Glucose concentration-dependent filamentation mediated via RAS-cAMP-PKA; in the presence of cAMP – no filamentation	(Timoumi et al. 2017b; Lesage et al. 2021)
Temperature		
38 ℃	twofold increase in the total cytosolic sugar content with concurrent substitution of MAN for TRE; 10 × increased concentration of arabitol	(Sekova et al. 2019)
	Drop in the storage and membrane lipid levels (by 35%), changes in their composition (like > threefold decrease in the sterols content and appearance of some sterol esters)	
	Increased activities of Sod2p and catalase Ctt1p	
	threefold increase in GSH level, tenfold increase in glutathione disulfide (GSSG) pool	
	Lipid bodies-nucleus-mitochondria continua – active migration of lipids	
37 ℃	Enlargement of mitochondria, enhanced number and enlargement of peroxisomes, formation of lipid and polyphosphate granules, formation of globular surface structures, enriched in silicone	(Biryukova et al. 2011; Arinbasarova et al. 2018)
	Dimorphic transition – unipolar growth, asymmetric division, large, polarly located vacuoles, and repression of cell separation after division	
37 ℃	Filamentation – elongation factor increased by 25%	(Kawasse et al. 2003)
42 ℃	Increased concentration of MAN (fourfold) and aKGA (threefold)	(Kubiak et al. 2021)
38 ℃	Induction of heat-shock proteins synthesis	(Sekova et al. 2021)
	Cell shrinkage – cofilin (F20856p)	
	Upregulation in thioredoxin (Trx1p), formate dehydrogenase (Fdh1p)	
	Upregulation of fructose-bisphosphate aldolase (Fba1p) enhanced TRE synthesis	
Thermotolerant strain BBE-18	Upregulation of amino acids synthesis, including Ala, Arg, Asn, Gln, and Met	(Qiu et al. 2021)
	Upregulation of phosphoglucomutase PGM1, pyruvate kinase PYK1, and erythrose reductase ER3	
	Key role of thiamine synthesis (E32681g, E35222g, A12573g, and F26521g) evidenced	
Dehydration		
Drying or freezing	Injury of the plasma membrane, changes in its fluidity and organization, lipids peroxidation, nucleic acids degradation, proteins dehydration and aggregation, cell wall disruption, causing cell shape alteration, and loss of cell integrity	(Pénicaud et al. 2014)
Oxidative agents		
0.5 mM H_2_O_2_	Accumulation of polyphosphate granules	(Biryukova et al. 2011)
0.5 mM H_2_O_2_	Globular structures on the cell wall surface – surface globules contain silicone	(Arinbasarova et al. 2018)
	Formation of the multi-layered plasma membrane and multiple membrane vesicles localized in proximity to the cell wall	
	Abrupt increase in cAMP and the following drop – activation of stress defense mechanisms	
1 mM paraquat hyperbaric air (3 or 5 bar) 50 mM H_2_O_2_	Lipid peroxidation	(Lopes et al. 2013)
	Increased GSH content	
	Upregulation of Glr1p and Sod2p activity	
	H_2_O_2_ – Ctt1p activity induction	
20 mM H_2_O_2_	Induced filamentation	(Kawasse et al. 2003)
Acetate	Decreased lipogenic potential	(Xu et al. 2017)
Toxic metals and chemicals		
Heavy metal ions	Filamentation	(Bankar et al. 2018)
	Formation of nanostructures on the yeast cell surfaces	
	Faulty cytokinesis	
Fe2^+^ and pH 3.0	Decreased abundance and activity of plasma membrane H + -ATPase Pma1p	(Cogo et al. 2020)
50 µM uranium	Increased cell size, irregular cell surfaces, membrane permeabilization	(Kolhe et al. 2020)
	Enhanced ROS generation, lipid peroxidation, transient RNA degradation, and protein oxidation	
	Upregulation of Sod2p activity, but not Ctt1p	
	Disappearance of vacuoles and other intracellular organelles	
50 µM uranium	Upregulation of transmembrane transporters (MFS and ATPase-coupled transmembrane transporter)	(Kolhe et al. 2021)
	Oxidative stress response – upregulation of GSH transferase/peroxidase, peptide-methionine (R)-S-oxide reductase and other oxidoreductases	
	DNA damage repair – mismatch repair, chromatin condensation (RCC1)	
	Structural rearrangements in cell wall – 1,3-β-glucanosyltransferase, chitin synthase	
	Cell division arrest at G2 phase – SMC2, SMC4, YCS4, YCG1, HOF1	
Ionic liquid	Damage of cell envelope – cavities, dents, and wrinkles	(Walker et al. 2019)
	Onset of restructuring within the cell wall and plasma membrane	
	Sterol biosynthesis was the only “lipid pathway” significantly perturbed	
	twofold increase in ergosterol content	
Osmo-active compounds		
Different effectors	Induction of HOG pathway: Sln1-Ypd1-Ssk1/2 – cytoplasmic functions of Hog1p: stabilization of stress-response transcripts Ubp3-driven turnover of specific transcription factors and/or RNA Pol II Sln1-Ypd1-Skn7 – nuclear functions of Hog1p: direct interaction with transcription factors and chromatin remodeling factors transcription of stress-response genes	
	A rapid and transient delay at various stages of the cell cycle	
	Depending on the osmo-active compound – induction or repression of rs-Prot synthesis	
0.5–1.0 M glycerol/glucose	Induction of ERY-dependent but HOG-independent osmoprotection mechanism	(Rzechonek et al. 2020)
6–9% NaCl	Decrease in cell size – rapid concentration of intracellular solutes, e.g., amino acids like proline, alanine	(Andreishcheva et al. 1999)
	Rapid action of cell membrane pumps and cytoskeleton	
3% NaCl = 4.21 Osm kg^−1^	Promoted synthesis of ERY – upregulation AKRs: Gcy12p, Tkl1p and Gcy15p	(Yang et al. 2015)
	Increased demand for energy – upregulation of a panel of proteins involved in glycolysis (Tpi1p), TCA (AcnAp; Mdh2p), and respiration (Cox4p, Mcr1p)	
	Onset of oxidative stress response – upregulation of Ctt1p, Sod2p Ahp1p, Sti1p, Hsp20p, Hsp12p	
	Upregulation of amino acids synthesis – Met6p, Shmtp	
	Downregulation of Gdh1p to decrease amino acids efflux to TCA	
	Adjustment of ions equilibrium – downregulation of membrane K + channel	
	Downregulation of protein synthesis (Tef1p, ribosomal 60S proteins L2 and L4, seryl-tRNA synthetase)	
	Upregulation of Prb1p vacuolar protease	
360 g L^−1^ sorbitol = 3 Osm kg^−1^	Upregulation of AKRs involved in polyols synthesis – 7 × higher concentration of MAN, 2 × ERY – Gcy13p, Gcy12p, A19910p, F24937p, D08778p	(Kubiak-Szymendera et al. 2021a, b)
	Downregulation of TCA and FA synthesis – threefold reduction in CA concentration	
	High increase in chaperoning and folding capacity – HSP20, STI1, FMO1, SSA6/7, and Ssa6p, Ssa8p, Hsp104p, Hsp90p, ER-localized E25696p, F00880p, and mitochondrial Hsp78p, Isu1p	
	No evidence for HOG1 upregulation at gene expression/protein abundance level, but upregulation of SKN7 and SKO1	
	Enhanced TRE synthesis – TPS1, TPS2, TPS3	
	Onset of oxidative stress response	
	Enhanced demand for energy – upregulation of D08602p, F24409p, D09933p, and transcriptional activation of TPI1	
	Sequestration of membrane channels and transporters – upregulation of cellular membrane invagination and endocytosis factors (Pil1p/Lsp1p), vesicle transportation (B14102p, F27379p), and the major vacuolar protease Prb1p	
	Downregulation of protein synthesis-related processes – Tef1p, ribosomal 60S proteins L2 and L4, and eight amino acid-tRNA synthetases, ribosome biogenesis (E31625p, F12661p) and biosynthesis of amino acids (Aro10p, Bat2p, Pro3p, CysK-Met25p, MetBp) increased amounts of uncharged tRNAs	
Over-synthesis of rs-Prots – strongly dependent on biochemical properties of the rs-Prots		
Over-synthesis of burdensome r(s)-Prots	Significant increase in the synthesis of stress-response molecule – MAN	(Korpys-Woźniak et al. 2020)
Over-synthesis of two demanding rs-Prots	Significantly increased demand for the substrate even at the reduced growth rate	(Gorczyca et al. 2022)
Over-synthesis of burdensome r(s)-Prots	Upregulated biological process – ion homeostasis	(Korpys-Woźniak and Celińska 2021)
	Increased abundance of vacuolar sorting and vacuolar proteases	
	Downregulation of ribosome biogenesis and rRNA processing	
Over-synthesis of highly synthesized and highly secreted rs-Prots	Increased energy demand – enhanced expression of genes localized to mitochondria	
	Significant upregulation of oxidative stress response genes	
	Downregulation of protein degradation, autophagy, and vacuolar protein sorting factors	
	Growth arrest phase (G1 phase)	
	Released ribosome assembly from inhibition	
Over-synthesis of two rs-Prots – larger and smaller	Accumulation of the larger protein’s transcript – indicating insufficient translation capacity	(Swietalski et al. 2020)
	Limitation of the smaller protein secretion level	
Over-synthesis of two complex rs-Prots	Accumulation of saturated FAs—marker of ER-stress	(Wei et al. 2019, 2021)
	Competition among synthesis/secretion of the protein and lipid synthesis	

## External stress factors

### Oxygen availability

The primary stress factor encountered in *Y. lipolytica* cultures is limited oxygen availability (OA), staying beyond the technical possibilities of laboratory or industrial equipment. Furthermore, spatial fluctuations in oxygen concentrations may range from 22 to 0% pO2 in well-mixed and stagnant zones of an industrial bioreactor, respectively (Oosterhuis and Kossen 1984). Likewise, a 64-fold difference between the maximum and minimum oxygen concentrations was predicted inside a pilot-scale bioreactor (Reuss et al. 1994). As *Y. lipolytica* is a strict aerobe, sufficient oxygen provision is of fundamental importance. We recently showed that limited oxygen transfer rate (OTR) strongly limits rs-Prot synthesis in *Y. lipolytica* (Gorczyca et al. 2020). The same observations were done for the production of organic acids and polyols (Rywinska et al. 2012; Li et al. 2017; Mirończuk et al. 2019). It was found that limited OA decreases the rate of all – transcription, translation, and secretion of a reporter protein – most probably due to insufficient provision of molecular machinery elements and energy (Gorczyca et al. 2020). Notably, the kinetics of protein synthesis was not directly related to the rate of biomass growth. On the other hand, it was observed that the ovoid morphotype was the more efficient producer rather than filamentous forms.

Comprehensive data on the molecular background underlying *Y. lipolytica*’s response to limited OA are still very scarce; however, some interesting results on its physiological reaction are available. It has been established that in poorly aerated environments, *Y. lipolytica* tends to grow in filamentous form, which is a recognized hallmark of ongoing cellular stress (Ruiz-Herrera and Sentandreu 2002). Bellou et al. (2014) evidenced that mycelia were mainly formed under low OA, whereas high aeration induced the growth in yeast-like form. Filamentation under low OA was accompanied by downregulation of lipid biosynthesis, as measured by the activity of ATP-citrate lyase (almost 14-fold lower) and malate dehydrogenase (nearly 23-fold decrease). Recent studies by Timoumi et al. 2017a, 2018) and Lesage et al. (2021) shed some new light on this phenomenon. Upon cultivation in batch mode, changes in OA (0 to 40%) triggered intermittent and completely reversible changes in growth rate, but filamentation was induced and progressively intensified (Timoumi et al. 2017a). In other words, restoring high OA after a period of anoxia restored maximum growth rate and re-consumption of metabolites, but did not reverse dimorphic transition. Over cultivation in glucose (GLUC)-limited continuous mode, *Y. lipolytica* cells grew constitutively in the yeast-like form at both fluctuating and low OA (pO2 ~ 2%; 75% of oxygen needs). Any filamentation could be observed solely upon the combination of low OA (pO2 0%) and the presence of GLUC at a concentration > 0.75 g L^−1^, which was a completely new observation. Interestingly, different frequencies of anoxia periods were analyzed, and it was noted that more filaments were formed when more frequent and shorter anoxia periods were simulated (12 × 5 min), even if the total time under oxygen deprivation was exactly the same (vs. 9 × 20 min or 3 × 1 h). The following research, the authors confirmed that dimorphic transition is actually dependent on GLUC availability, also, when filamentation is induced by pH (Timoumi et al. 2017b). Molecular background of that observation was studied (Lesage et al. 2021) and will be discussed in the following section of this review.

### Acidity – pH

While pH is relatively easy to stabilize in bioreactor cultivations, local fluctuations in the culture acidity are known to occur. In practice, it is desirable to conduct industrial-scale bioprocesses with *Y. lipolytica* under very low pH (3.0) which limits the risk of undesired microbiota development, but is at the lower margin of pH tolerance range for *Y. lipolytica*. As recently studied, the maximal linear growth for the reference strain W29 was observed at pH 5.5, and no growth at pH below 3.0 and above 10.5 (Sekova et al. 2019). Even though the growth of *Y. lipolytica* is maintained within such a wide pH range, its metabolic capacity differs substantially depending on ambient acidity (Sassi et al. 2017). It is known that even under extreme acidic environmental conditions (pH 3.0), the intracellular pH of a eukaryotic cell can still be maintained near neutrality (pH ~ 6.8), but the metabolic effort required to maintain the homeostasis is significant (Madshus 1988). It was shown that under pH 3.0, the abundance and activity of plasma membrane H^+^-ATPase Pma1p (B22066g) is significantly higher than at pH 4.5 or 6.0 (when it pumps H^+^ ions inwardly) (Cogo et al. 2020). Pma1 which generates the proton gradient and drives the active transport of nutrients by H^+^-symport is the most abundant protein in the yeast plasma membrane (Ferreira et al. 2001). It is also a major consumer of cellular ATP and has been estimated to consume at least 20% of ATP in the cell. Some insights into the physiological and molecular background of non-optimal-pH-response in *Y. lipolytica* are available.

In yeast, pH sensing/signaling is typically operated by a PAL1-4-RIM101 pathway (Bahn and Jung 2013; Day and Quinn 2019). A Rim101p (B13640g) and four Pal1-4p (B06710g, F12397g, D19162g, B22814g) homologues in *Y. lipolytica* were found to be implicated in the control of alkaline/acidic serine protease (Xpr2p/Axp1p) synthesis under near-neutral/acidic conditions, as well as in mating and sporulation (Lambert et al. 1997). It was suggested that *RIM101* transcription is auto-regulated in a pH-dependent manner. A developed model, based on similarities found in the other fungi, presumed that ambient neutral-alkaline pH is sensed by a Pal1-4p cascade which transduces the signal and makes Rim101p protein a substrate for an unidentified protease that removes 60% of the polypeptide from its C-terminus. The proteolytic cleavage is required for its activation. Such an active form turns on/off transcription of genes expressed at alkaline (*XPR2*)/acidic (*AXP1*) pH. The synthetically truncated form of Rim101p activated *XPR2* expression regardless of the pH and of the status of the *PAL* genes; and repressed *AXP1* transcription at acidic pH. In the following study, Rim101p was found to bear a DNA-binding ability and regulate expression of genes in a pH-dependent manner (Madzak et al. 1999). Nevertheless, *Δrim101* mutants did not exhibit any growth defects or changes in yeast-to-hyphae transitions over a broad range of ambient pH values (pH 3.5–8.0) (Lambert et al. 1997).

Sekova et al. (2019) studied the adjustment of intracellular sugar and lipids pools in response to alkaline conditions (pH 9.0). As observed, under exposure to pH 9.0, the intracellular sugar amounts declined by 25%. Qualitatively, the main fraction was represented by mannitol (MAN; nearly 90%), followed by arabitol. No GLUC was detected. Surprisingly, the common stress response molecule – trehalose (TRE), was not synthetized upon exposure to the alkaline pH. Storage lipids as either lipid bodies or membrane lipids were decreased by ~ 30% and 36%, but the level of saturated fatty acids (sFAs) in mitochondria was elevated. The alkaline pH triggered a significant increase in superoxide dismutase activity (Sod2p; B08921g) by 5.8-fold and halved reduced glutathione (GSH) concentration, the hallmarking onset of oxidative stress. In the following study, Sekova et al. (2021) implemented comparative proteomics to reveal the molecular background of the alkaline stress response. *Y. lipolytica*’s proteome under pH 9 (vs. 5.5) was characterized by significant changes within cell wall proteins abundance, an increase in chaperoning capacity, substantial changes in mitochondria activity, as well as a shift in the central carbon metabolism toward pyruvate rather than gluconeogenesis. More specifically, several proteins were found to be unique or highly induced under that stress condition, like members of the heat shock protein (HSP) family (C03443p), mitochondrial chaperones (C17347p, F02805p), nascent polypeptide association complex (F08393p), proteins involved in ubiquitination – E3 ubiquitin ligase (A10879p) and endopeptidase activator (B09339p), glycolytic glyceraldehyde-3-phosphate dehydrogenase (GAPDH; C06369p) and triose phosphate isomerase (Tpi1p; F05214p), mitochondrial proteins – malate dehydrogenase (D16753p) and VDAC porin (F17314p), NADPH dehydrogenase (B07007p), as well as calcium binding protein (E03388p). A highly upregulated under pH 9.0 rotamase (C10230p), inflicted in cytoplasmic folding of polypeptides, is known to interact with the histone deacetylation complex (HDAC; B20262p), which regulates transcription rate. Concomitant activation of TPI1 and GAPDH hallmarks shift in carbon flux toward pyruvate and tricarboxylic acid cycle (TCA). Simultaneous induction of mitochondrial malate dehydrogenase and VDAC designates the key role of mitochondria in metabolic readjustment and active participation in stress response. Due to its key role in mitochondria operation, regulation of respiration, ROS homeostasis, and yeast stress tolerance, VDAC was deemed as one of the key proteins responding to the alkaline pH. The VDAC porin closely interacts with the other proteins of known pore activity (F31207p, B10362p, and A07084p), identified in the proteome, involved in import of proteins into mitochondria. Several of the differently abundant proteins (DAPs) were downregulated or completely disappeared from the proteome of pH 9.0-treated cells. These included proteins of oxidative phosphorylation (D2202p, E19723p, E10144p), the key cell wall mannoprotein Pir1p (B20306p) and B03564p involved in cell wall biogenesis, 60S ribosomal ubiquitin (Q6C2D7), and thioredoxin (Trx1p; F01496p). The decrease in ubiquitin was associated with some disturbances in the ribosomes’ structural organization.

Complementary research on proteomic readjustment of *Y. lipolytica* to acidic pH (4.0 and 3.0) was conducted by Guo et al. (2016). The cells from the late-exponential growth phase grown at pH 5.5 were exposed to stressful environmental conditions. Such a strategy was known to enhance the synthesis of the target molecule of that study aKGA (alpha ketoglutaric acid). Comparative proteomics was conducted to get an insight into the molecular background underlying the improved production. Differentially abundant proteins were grouped into functional categories, of which several were directly functionally and structurally localized to mitochondria, like the enriched group of the TCA proteins (Cit1p/E00638g, Pda1p/F20702g, Pdb1p/E27005g, Mdh1p/D16753g, Icl1p/C16885g, Kgd2p/E16929g, and Acs2p/F05962g). Proteins acting upstream from TCA, involved in glycerol (GLY) assimilation feeding the TCA (Pgk1p/D12400g, Gut2p/B13970g, Eno2p/F16819g, Tpi1p/F05214g, Tdh3p, Fba1p/E26004g, and Pck1p/C16995g), and downstream from TCA, involved in the mitochondrial electron transport chain and ATP synthesis channeling the TCA’s products (Nuamp/D05467g, Nuemp, Nufmp/E23089g, Qcr2p/F08613g, Atp1-2–3-7p/D12584g, Cdc48p, and Afg1p/D00649g), were all upregulated, further supporting the statement on the key role of mitochondria in response to the acidic pH. Likewise was the significant upregulation of mitochondrial porin (Por1p/F17314g), involved in small molecular transportation between cytoplasm matrix and mitochondrial matrix. Collectively, it was postulated that intracellular proton extrusion across the mitochondrial inner membrane by electron transport chain was the major mechanism contributing to the pH homeostasis maintenance upon exposure to the acidic pH. However, the leakage of protons from the electron transport chain led to an intracellular outburst of reactive oxidative species (ROS) and oxidative stress. To counteract this, the cell increased synthesis of amino acids (Aat2p/B02178g, Leu1p/B01364g, Gcv2p, Sam2p, Met6p/E12683g, Ilv5p/D03135g, Ses1p/F02629g, Gat1p/F17886g, and Shm1p/D22484g), molecular chaperones (Kar2p/E13706g, Sse1p/E13255g, and Ssa4p/D22352g), superoxide dismutase (Sod2p/B08921g), and the target aKGA, which are all known to play a role in scavenging ROS. The proposed mechanism is consistent with findings by Cogo et al. (2020), showing that acidification of external pH is accompanied by enhanced H^+^ efflux through the operation of plasma membrane H^+^-ATPase Pma1p (B22066g). Transportation of carboxylic acids exploits this preformed membrane H^+^ gradient. Operation through this mode explains the enhanced synthesis of organic acids by *Y. lipolytica* under acidic pH. Finally, it was proposed that aKGA is also a stress response molecule, which acts as a pH titrant to buffer cellular matrix and as an antioxidant, protecting the cells against ROS. Consistently, aKGA synthesis was significantly enhanced upon exposure to heat stress (42 ℃ for > 150 min), demonstrating its universal implication in stress-response (Kubiak et al. 2021).

In their research, Timoumi et al. (2017b) and Lesage et al. (2021) investigated the effects of ambient pH and mode of culturing on *Y. lipolytica* stress response manifested as filaments formation (used as a marker of stress). In the former study (Timoumi et al. 2017b), it was observed that in batch cultures, a shift in pH toward either acidic (pH 4.5) or neutral (pH 7.0; termed alkaline stress) induced filaments formation without detectable effect on the macroscopic behavior of cells (growth rate, biomass, oxygen, and carbon source utilization). In contrast, in continuous cultures, at controlled growth rates (from 0.03 to 0.20 h^−1^) even close to the maximum growth rate of the strain (0.24 h^−1^), only ovoid cells were observed, irrespective of the pH. It was inferred that pH shifts induced mycelial growth during batch cultivations due to the presence of residual carbon sources. In contrast, no dimorphic transition was triggered in GLUC-limited chemostat cultures, irrespective of growth rate or pH. This hypothesis was further tested in the following study (Lesage et al. 2021), where the impact of residual GLUC concentrations on the induction of the dimorphic transition in response to pH stress was investigated in detail. Based on previous reports on the opposite action of two signaling pathways, MAPK (mitogen-activated protein kinase) and cAMP-PKA (cyclic-AMP-dependent protein kinase A), in the regulation of dimorphic transition in *Y. lipolytica* (Ruiz-Herrera and Sentandreu 2002; Cervantes-Chávez and Ruiz-Herrera 2006, 2007; Cervantes-Chávez et al. 2009), intracellular cAMP levels measurement and its supplementation were inflicted in that research, in addition, the pH stimuli. As stemmed from those previous literature data, the MAPK pathway promotes mycelial growth, while the cAMP-PKA pathway is required for growth in the ovoid morphotype. Primarily, using an accelerostat approach (increasing growth rate to modulate residual GLUC concentration), it was possible to determine the threshold GLUC level around 0.35–0.37 mg L^−1^, above which filamentation could be induced by the environmental stimuli. The filamentation was titratable, as the increase in the residual GLUC levels intensified the formation of the elongated morphotype. It was hence ultimately determined that dimorphic transition in *Y. lipolytica* is much more controlled by a sugar signaling pathway rather than by pH (Timoumi et al. 2017b) or OA (Timoumi et al. 2017a). Three different GLUC signaling pathways are known to operate in yeast: (1) the RGT2/SNF3 (in *Y. lipolytica*—C06424g/C08943g) GLUC induction pathway, (2) the SNF1/MIG1 (D02101g/E07920g) GLUC repression pathway, and (3) the RAS-cAMP-PKA pathway. Based on previous evidence, the specific role of cAMP levels on dimorphic transition in response to pH perturbations was studied using a microfluidic culturing system. As observed, the exogenous addition of cAMP abolished the mycelial growth of *Y. lipolytica*, even with GLUC concentrations exceeding the threshold level. It was thus finally concluded that the morphological response of *Y. lipolytica* to pH (and OA) perturbations was different depending on the residual GLUC concentration, which was most probably mediated via the cAMP-PKA-type signaling pathway.

### Temperature

As the majority of the other yeast species, *Y. lipolytica* grows well at temperatures close to 28–30 ℃, showing a preference toward slightly psychrophilic conditions. The reference W29 strain was able to grow in a wide range of temperatures from 20 to 40 ℃ when pH was maintained at 5.5, showing maximal growth at 29 ℃ (Sekova et al. 2019). No growth was observed at > 40 ℃, and biomass formation was substantially reduced at 38 to 40 ℃.

With the aim to study the effects of elevated temperature on *Y. lipolytica* cells, (Sekova et al. 2019) tracked changes in the intracellular pools of carbohydrates and lipids, and the activity of the major enzymatic ROS detoxifiers. Under optimal temperature (29 ℃), MAN was the major cytoplasmic sugar alcohol, and any changes to pH did not result in changes to lipid composition, provided that temperature was stably maintained. Increased temperature (38 ℃) led to over a twofold increase in the total cytosolic sugar content with concurrent substitution of MAN for TRE (reaching approx. 70% of total sugar content), and a tenfold increase in arabitol level (mounting up to 25% of the total sugar content). It was not surprising, as TRE is known to play a key role in *Y. lipolytica* cells protection against heat shock. Disruption of *TPS1* (E14685g) drastically slowed growth at 35 ℃ (Flores et al. 2011). On the other hand, in another study, after exposure of *Y. lipolytica* strain to heat shock at 42 ℃ for > 150 min, MAN and aKGA synthesis were enhanced by over fourfold and nearly threefold, respectively (Kubiak et al. 2021). The elevated MAN concentration does not necessarily contradict observations by Sekova et al. (2019), as on the one hand, the treatment conditions differed in severity (38 ℃ for 1 h vs. 42 ℃ for 2.5 h), but foremost, it is plausible that intracellular accumulation of TRE was accompanied by an abrupt extrusion of MAN (and aKGA) to the medium, which was not studied by Sekova et al. (2019).

Furthermore, in the studies by Sekova et al. (2019), the exposure to the elevated temperature was associated with a substantial drop in the storage and membrane lipid levels (by 35%), changes in their composition (like > threefold decrease in the sterols content and appearance of some sterol esters), and increased unsaturation degree of FAs. Exposure to 38 ℃ increased activities of Sod2p and catalase Ctt1p (E34749g/E34265g) by over 12-fold, GSH level was elevated threefold, and glutathione disulfide (GSSG) pool by tenfold. Transmission electron microscopy revealed the formation of lipid bodies-nucleus-mitochondria continua, hallmarking the active migration of lipids between the organelles. Heat shock also triggered a 15% decrease in cell size. Correspondingly, multiple structural changes in *Y. lipolytica* cells were observed after mild heat shock treatment (37 ℃, 60 min) (Biryukova et al. 2011; Arinbasarova et al. 2018). Those structural modifications comprised enlargement of mitochondria, enhanced number and enlargement of peroxisomes, formation of lipid and polyphosphate granules, as well as numerous globular surface structures, enriched in silicone. Another study demonstrated that following a thermal treatment at 37 ℃ for 1 h, the cells elongation factor (ratio between hyphal length and hyphal width) was increased by 25%, indicating other structural changes in response to the heat stress (Kawasse et al. 2003).

In the following study, insight into the molecular background of the heat stress response in *Y. lipolytica* was obtained by comparative proteomics (Sekova et al. 2021). The adopted thermal treatment (38 ℃, 1 h) led to changes in the cell wall proteins, including the disappearance of mannoprotein (Pir1p, B20306p) and 1,3-beta-glucanosyl-transferase (B03564p), as it was observed under the alkaline conditions (Sekova et al. 2021). On the other hand, the elevated temperature was associated with the unique occurrence of several heatshock proteins like E35046p, D22352p, CPAR2_700380, serving as cytoplasmic chaperones. Macroscopically observed cell shrinkage was associated with the upregulation of cofilin (F20856p), which is the main regulator of actin dynamics. Elevated temperature led to upregulation in thioredoxin (Trx1p; F01496p) and formate dehydrogenases (Fdh1p; B22506p). The former was directly associated with the accompanying increase in ROS levels (also hallmarked by increased activity of Sod2p and Ctt1p (Sekova et al. 2019)). For the latter, formaldehyde can form due to the catabolism of amino acids and nucleotides, but also from DNA demethylation. Upregulation in Fdh1p suggests induction of catabolic processes related to scavenging damaged proteins and lipids, as well as gene expression readjustment by chromatin remodeling under the conditions of stress. Macroscopically observed synthesis of TRE was hallmarked in the stress proteome by upregulation of fructose-bisphosphate aldolase (Fba1p; E26004g) participating in glycolysis and gluconeogenesis, required for TRE synthesis. Combination of the thermal and alkaline stress conditions (pH 9.0, 38 ℃) led to cross-adaptation, hallmarked as a change in the chaperone compositions and readjustment of the proteomes for redox adaptation, catabolic processes, and oxidative phosphorylation (increase in pyruvate dehydrogenase, ATP synthase subunit, GAPDH; identification of new unique spots for carbonyl reductases). Interestingly, GTPase cytoplasmic elongation factor 1 alpha Tef1p (C09141g), a protein required for ribosomes assembly, was uniformly upregulated upon heat shock, implemented alone or in combination with the alkaline stress. Its abundance indicates on ongoing synthesis of new polypeptides, which, most probably, account for long-term adaptation mechanisms.

In reference to the upregulation of Fdh1p in response to heat stress (Sekova et al. 2021), and its implication in DNA demethylation, the impact of stress conditions on DNA methylation level in *Y. lipolytica* was studied recently (Kubiak-Szymendera et al. 2021a). Two types of stress factors were implemented in that study – repeated subculturing and heat shock (42 ℃ 1 h). 5-Methyl-cytozine (5mC) level was determined by immunoassay and NanoPore whole-genome sequencing. Based on the immunoassay results, 5mC was found to occur at 0.1 to 0.5% frequency, which was consistent with results obtained for this species with the other analytical methods (Tang et al. 2017). Interestingly, the 5mC level was not different depending on the implementation of the heat shock or not, but it differed significantly depending on the cycle of subculturing. It was observed that DNA methylation level decreased with each subculturing cycle and was also significantly decreased when the cells entered the stationary phase of growth compared to the log phase. The decrease in 5mC % was also accompanied by increased filamentation frequency, implying an ongoing stress response. Altogether, those results suggested that while the implemented heat-shock conditions did not impact DNA methylation level, but repeated subculturing awoke stress response and long-term adaptation mechanisms. Interesting research into short- and long-term adaptation to osmotic stress in *Candida albicans* is available (You et al. 2012), but falls beyond the scope of this review.

An adaptive laboratory evolution approach was used to develop a thermotolerant *Y. lipolytica* strain able to synthesize erythritol (ERY) at elevated temperatures (Qiu et al. 2021). With that practical aim in mind, the authors studied the background of *Y. lipolytica* adaptation to increased temperatures by global transcriptome profiling. First, a thermotolerant strain BBE-18 was obtained after 11 months of continuous cultivation and selection. The resultant strain maintained the same specific growth rate at 35 ℃ as its parental strain at 30 ℃. Comparative transcriptomics revealed that many genes involved in the central carbon metabolism were upregulated in the evolved strain, including phosphoglucomutase *PGM1* (B02728g), pyruvate kinase *PYK1* (F09185g), and erythrose reductase (*ER3;* either B07117g/F18590g/D07634g/C13508g (Janek et al. 2017; Cheng et al. 2018)). On the other hand, it was observed that at 37 ℃ transcription level of aKGA dehydrogenase complex *KDH* (*KGD1*/E33517g, *KGD2*/E16929g, *LPD1*/D20768g; (Holz et al. 2011)) was significantly decreased in the evolved strain, suggesting decreased synthesis of ATP. Significant upregulation was seen in the transcription of many genes involved in amino acids synthesis, including alanine, arginine, asparagine, glutamine, and methionine. Knowing that alanine is one of the main compatible solutes, arginine and asparagine are known to exert a beneficial effect on cell resistance to stress, and that glutamine is an important amino donor, it was inferred that this readjustment in amino acid metabolism was a significant element of the acquired thermotolerance. Subsequently, considering the key role of thiamine in multiple metabolic processes that were deregulated in *Y. lipolytica* upon exposure to the elevated temperature, it was postulated that higher provision of thiamine may impact many individual metabolic processes cooperatively promoting thermotolerance without modifying hundreds of genes. To this end, four genes directly related to thiamine synthesis (E32681g, E35222g, A12573g, and F26521g) were overexpressed individually or in combination, and the resultant strains were tested under heat stress. The modified strain grew significantly better under elevated temperature, and one of the underlying factors contributing to improved performance was the increased ATP pool.

With the aim to enhance thermotolerance of *Y. lipolytica*, Wang et al. (2020) overexpressed the *RSP5* gene from *S. cerevisiae* encoding E3 ubiquitin ligase. E3 ubiquitin-protein ligase accepts ubiquitin from an E2 ubiquitin-conjugating enzyme in the form of a thioester and then directly transfers the ubiquitin to targeted substrates. It is also involved in intracellular trafficking of the general amino acid permease Gap1p as well as other cell surfaces proteins like Pma1p and Ste2p. The protein is involved in the expression of heat-shock element-mediated gene expression, nitrogen starvation GLN3-dependent transcription, as well as actin cytoskeleton organization and dynamics. The *RSP5*-overexpression strain resultant strain could grow well up to 35 ℃ and retain an efficient ERY production capacity at 33 ℃. Its survival rate after being heat-shocked at 45 ℃ for 1 h was also significantly improved over the parental strain. The expression level of *RSP5* was studied under exposure of the recombinant strain to different temperatures (30 ℃, 33 ℃, 36 ℃, 39 ℃, and 42 ℃) for 4 h. It was observed that any increase in the ambient temperature above 30 ℃ led to a 2- to threefold higher expression level of the target gene, even though the promoter and the reference gene remained the same. In contrast, a recombinant gene transcript level was found to be the highest when the temperature was decreased to 20 ℃ when compared to 31 ℃ or 42 ℃ (Kubiak et al. 2021). The latter treatment (42 ℃) led to a slight decrease below the transcript’s level determined for 31 ℃, but it also severely reduced viable cells counts, which was not the case for the *RSP5*-overexpression strain, even when treated with more severe condition (45 ℃ 1 h). Consistently with Wang’s data (Wang et al. 2020), a heterologous gene was expressed at equal or higher levels at 30 ℃ when compared to 25 ℃ (Korpys-Woźniak et al. 2021).

Large changes to ambient temperature in extreme cases can lead to complete evaporation of water (drying) or its sequestration in the form of crystals (freezing). In both cases, water is not available for biological processes. Biological consequences of dehydration are dramatic to the cell, comprising severe injury of the plasma membrane, changes in its fluidity and organization, lipids peroxidation, nucleic acids degradation, proteins dehydration and aggregation, and cell wall disruption, causing cell shape alteration and loss of cell integrity. The effects of such environmental stress were studied at physiological and biochemical levels in *Y. lipolytica* (Pénicaud et al. 2014). As in the case of resistance to oxidative stress (Biriukova et al. 2006), stationary-phase cells were more resistant to dehydration by drying when compared to log-phase cells, as they showed higher cultivability after revival. The same relates to cells that were suspended in TRE solution prior to the treatment. Drying of the cells that were harvested at log-phase brought 98% mortality, when no TRE-medium protection was provided. Suspension of the cells in TRE solution prior to the treatment limited mortality to 34%, while harvesting in stationary phase resulted in 79% survival (21% mortality). TRE was proven efficient in securing stability of intracellular proteins, as demonstrated using esterase activity as a reporter, which is consistent with the known role of TRE in replacing water removed during dehydration. On the other hand, neither harvesting at stationary-phase nor treatment with TRE could protect the cells from DNA damage after drying/rehydration. The same was observed for cell wall damage after drying/rehydration. Freezing/thawing of any growth-phase cells did not bring any substantial changes to biochemical composition, viability, and cell membrane stability. Membrane permeabilization measured by PI staining was significantly correlated with cultivability loss, highlighting the crucial role of membranes in the resistance to dehydration/rehydration. FT-IR analysis highlighted several biochemical traits of *Y. lipolytica*, as for example that cells harvested in the log phase contained more nucleic acids which was caused by increased nucleus-to-cytoplasm ratio and/or by a greater amount of measurable nucleic acids due to less condensed chromatin during replication, or that stationary-phase cells have thicker cell walls and contain longer lipid chains than log-phase cells (Pénicaud et al. 2014).

### Oxidative agents

Cellular oxidative stress can be awakened by numerous extrinsic and intrinsic factors. For example, oxidative stress response accompanies the cell’s reaction to perturbations in pH or temperature, as discussed above. Likewise, over-synthesis of heterologous secretory protein frequently leads to endogenous oxidative stress (to be discussed hereafter).

*Y. lipolytica*’s cellular response to oxidative stress induced by H_2_O_2_ (50–100 mM) or the superoxide-generating substances, menadione (0.25–0.5 mM) and juglone (0.025–0.05 mM), was studied for cells harvested at the exponential or stationary phase of growth (Biriukova et al. 2006). It was confirmed that the stationary-phase cells were generally more resistant to the action of the oxidative agents. It was shown that the permeability of stationary-phase cells to H_2_O_2_ was five times lower than that of exponential-phase cells. This observation was associated with increased levels of ergosterol in the stationary-phase cell membranes, which is a known factor responsible for permeability. In addition, GSH reductase (Glr1p; E18029g) activity in stationary-phase cells was found to be more than twofold higher than in the exponential-phase cells, without any treatment. Also, it was established that pretreatment with low concentrations of the chemicals provided the cells with increased (cross-)resistance. The intrinsic factor contributing to this was the induction of Sod2p/Ctt1p activity in the pretreated cells, so at the moment of the actual stress implementation, the cells were already secured with active antioxidant enzymes. Subsequently, Biryukova et al. (2011) analyzed ultrastructural changes in *Y. lipolytica* cells following exposure to 0.5 mM H_2_O_2_ for 1 h. It was shown that the yeast cell responds to the treatment with an accumulation of polyphosphate granules (suggested to be GLUC-diphosphate). Moreover, the exposure triggered the formation of globular structures of unknown nature on the cell wall surface. The following research on the modification of cellular envelope and intracellular structures in response to mild oxidative stress revealed that those surface globules contain silicone (Arinbasarova et al. 2018). Those studies also evidenced the formation of the multi-layered plasma membrane and multiple membrane vesicles localized in proximity to the cell wall. It was suggested that the unusual structure of the cell membrane and appearance of the extracellular vesicles hallmarked active remodeling ongoing in the stressed cells, and/or intensified transportation of molecules across the cell wall (including proteins, lipids, pigments, polysaccharides, and RNA). The exposure to the stress was also accompanied by an abrupt increase in cAMP levels which rapidly decreased after several minutes following the treatment implementation. A high cAMP level activates the PKA pathway which inhibits the expression of genes involved in defensive mechanisms. The initial spike in cAMP level (within the first 5 min) was not explained, but the following drop was associated with the activation of stress defense mechanisms.

Oxidative stress severity in response to superoxide- (paraquat at 1 mM, and hyperbaric air at 3 or 5 bar) and peroxide- (H_2_O_2_ at 50 mM) stress induction in *Y. lipolytica* was investigated by Lopes et al. (2013). The exponential-growth cells were subjected to the stress conditions for 3 h suspended in either rich medium of PBS buffer, followed by determination of the cells’ survival, the activity of the main ROS scavenging enzymes, and malondialdehyde content, which is a marker of lipid peroxidation. The highest effect on lipid peroxidation was observed after the treatment with H_2_O_2_ and paraquat, while the hyperbaric air had a smaller effect. The highest GSH content was detected, followed by the treatment with paraquat, with nearly no effect caused by H_2_O_2_. Glr1p activity was particularly upregulated when the treatment was executed in a buffer, while in the presence of a rich medium, the activity was not upregulated. In this case, the highest upregulation was seen upon treatment with paraquat and hyperbaric air. The latter stress factor at 5 bar was the most efficient inducer of Sod2p activity. In contrast, Ctt1p activity was the most induced by H_2_O_2_ treatment. Interestingly, those data demonstrated that *Y. lipolytica* grows significantly better (in terms of biomass accumulation) under increased air pressure, which is related to its high demand for OA. Another study showed that exposure of exponential-growth cells to H_2_O_2_ at 20 mM significantly induced filamentation of the cells (Kawasse et al. 2003).

Evidence of the direct link between oxidative stress and cell morphology (and lipid metabolism) was also provided by Xu et al. (2017), who aimed at improving lipid storage capacity in *Y. lipolytica* via modification of oxidative stress response. First, it was observed that lipogenic potential was reversely correlated with the level of reactive oxygen and aldehyde species, which level was modulated by supplementation with acetate (ROS generator) or MAN (ROS scavenger). Knowing that oxidative stress response was engineered by overexpression of native *SOD2* (B08921g), *GLR1* (E18029g), and GSH peroxidase (E02310g) to scavenge the excess ROS and maintain redox homeostasis, which improved lipid titer and oil content. The further strategy implemented in that study comprised overexpression of a broad substrate range of aldehyde dehydrogenase (*ALDH*) from *E. coli*, which was efficient in terms of reactive oxygen and aldehyde species elimination, and further improved the lipogenic potential of the yeast. Coupling *ALDH* overexpression with GLUC-6-phosphate dehydrogenase (*ZWF1*) from *S. cerevisiae* brought further improvements. The beneficial effect was even more enhanced upon combinatorial cloning of the heterologous genes with the oxidative stress defense genes (*SOD2*, *GLR1*, GSH peroxidase) and thioredoxin (*TRX1*; F01496g). It was observed that the original strain generated a considerable fraction of pseudohyphal and mycelial morphologies, while the ROS-deprived descendant (*aldh* +) developed round, isolated, and singular cells and contained a large fraction of oil droplet. Challenging the latter strain with 1 mM H_2_O_2_ led to elongated and pseudohyphal morphology and fewer oil droplets, evidencing that oxidative stress is closely intertwined with cell morphology and lipid metabolism. Apart from the practical outcome in the form of enhanced oleogenic potential of the strain, that study provided further insight into molecular bases of oxidative stress in *Y. lipolytica*.

Resistance to oxidative stress (50 mM for 1 h) was severely impeded in the *Δmhy1* strain (Konzock and Norbeck 2020). Mhy1p (B28150g) belongs to Msn2/Msn4-family C2H2-type zinc finger transcription factors (Hurtado and Rachubinski 1999; Wu et al. 2019) and has been suggested to constitute a functional homolog of the stress-responsive transcription factors Msn2p/4p in *S. cerevisiae*, the deletion of which causes severe stress sensitivity. It was evidenced that Mhy1p plays a critical regulatory role in various biological processes, such as dimorphic transition, lipid biosynthesis, amino acid and nitrogen metabolism, and cell cycle (Hurtado and Rachubinski 2002). From among three deletant strains (*Δhoy1*/A19214g; *Δcla4*/C31453g), only *Δmhy1* consistently formed an ovoid morphotype under any conditions tested. Interestingly, the *Y. lipolytica Δmhy1* strain survived equally well, or even better than the wild-type strain, under carbon and nitrogen starvation conditions, respectively, which suggests that Mhy1p is implicated in the nitrogen sensing/signaling pathway.

### Toxic metals and chemicals

Using cellular morphology (expressed in elongation factor ratio) as an indicator of the stress response, Bankar et al. (2018) studied the impact of different heavy metals (Cu (II), Zn (II), Pb (II), Cr (III), Co (II), Ni (II), Cr (VI), Cd (II), and As (V)) on *Y. lipolytica* cellular stress severity. Based on a comparison with the control conditions, exposure to 0.5 mM Pb (II), 0.5 mM Zn (II), 0.5 mM Cr (III), and 0.1 mM Cr (VI) contributed to the highest filamentation degree and formation of nanostructures on the yeast cell surfaces. Those observations were consistent with what was observed by Biryukova et al. (2011) and Arinbasarova et al. (2018) under simulated oxidative stress. Specifically, yeast-to-mycelium transition was observed in the presence of Cr (VI) and Pb (II), significant elongation of the cells was observed in the presence of Zn (II) and Cd (II) ions, while multipolar buds were observed in the presence of Cr (III), As (V), Ni (II), and Cd (II). Pb (II) at 1.0 mM and 2.0 mM promoted the formation of nanostructures on cell surfaces, while Cr (VI) at 0.1 mM led to faulty cytokinesis. Since hyphae were not observed in the presence of some metals such as Cu (II), Ni (II), As (V), and Cd (II), it was concluded that hyphae formation was metal-specific. It would be highly interesting and desirable to study the expression level of genes known to be involved in the dimorphic transition, especially those recently identified via massive mutation and genome sequencing studies (Pomraning et al. 2018), under exposure to the hyphae-inducing heavy metals indicted by Bankar et al. (2018).

The interplay between acidic pH (3.0, 4.5 vs. 6.0) and supplementation with iron ions (1 mM and 2 mM FeSO_4_) on stress response and H^+^ fluxes in *Y. lipolytica* was addressed by Cogo et al. (2020). Upon combination of the two stress factors, it was clear that a pH of 3.0 had any (negative) impact on the *Y. lipolytica* growth rate. Treatment with FeSO_4_ did not overcome this growth limitation but significantly decreased filamentation (deemed as a stress response) under pH 4.5 and 3.0. As shown by a scanning ion-selective electrode technique, in the absence of Fe^2+^, inward H^+^ fluxes were identified at pH 4.5 and 6.0 correlated with a pH increase at the cell surface (pH ~ 7.5) and their elongation. Conversely, a remarkable H^+^ efflux was observed at pH 3.0, related to the extracellular microenvironment acidification and predominant (but not sole) growth in the ovoid morphotype. Supplementation with Fe^2+^ ions enhanced H^+^ influx at pH 4.5/6.0 and inhibited H^+^ efflux at pH 3.0. It was evidenced that supplementation with iron led to decreased abundance and activity of plasma membrane H^+^-ATPase (Pma1p; B22066g), which contributed to the observed changes in H^+^ fluxes.

With a practical aim in mind of using yeast in bioremediation, Kolhe et al. (2020) studied the impact of uranium exposure (10–1000 µM of uranyl carbonate) on *Y. lipolytica* cells. Starting from 50 µM uranyl carbonate concentration, the growth of the yeast was limited, and over 700 µM was completely abolished. Cells exposed to 50 µM of uranium exhibited numerous structural changes, like increased cell size, irregular cell surfaces, or membrane permeabilization, observed as leakage of nucleic acid and proteins. Enhanced ROS generation and lipid peroxidation, transient RNA degradation, and protein oxidation were all observed in the exposed cells. Upregulation of Sod2p activity, but not Ctt1p, was detected for the cells subjected to 50 µM uranyl carbonate, which was completely abolished under higher uranium concentrations, probably due to too high damage caused to the cells. Prolonged exposure to 50 µM of uranium led to the complete disappearance of vacuoles and other intracellular organelles. Transmission electron microscopy allowed us to visualize the process and kinetics of uranium sorption and bioprecipitation. Short needle-like crystalline precipitates of uranium phosphate were localized intra- and extracellularly, proving successful biomineralization. In the following study, whole transcriptome profiling was adopted to reveal the molecular background of the macroscopically observed phenomena (Kolhe et al. 2021). Altogether, 56 differentially expressed genes (DEGs) with significant upregulation or downregulation were identified. Expectedly, the highly upregulated genes were identified to be involved in transport, DNA damage repair, and oxidative stress response. Among the most upregulated transcripts, a set of transporters bearing a major facilitator superfamily (MFS) domain was identified (C01001g, D02014g, F19492g, C08965g, and D00407g). MFS transporters are regulated by stress-responsive transcription factors and were found to be responsible for resistance to toxic compounds. So their putative role as uranium metal exporters was inferred. A similar role was assigned to upregulated ATPase-coupled transmembrane transporter E20016g, and P-loop-containing nucleoside triphosphate hydrolase B12188g involved in active transport. ATPases are known to play a significant role in the efflux of heavy metals and in protecting cells from damage. Uranium exposure enhanced transcription of *GPR1*/*FUN34* (C23298g) genes implicated in glyoxylate pathway regulation and acting as acetate transporter, as well as several genes involved in carbon metabolism, including acyl-CoA N-acetyltransferase (D06391g), thiamine pyrophosphokinase THI80 (E21351g), proteases (F05940g, E05423g), or hydrolases of different specificity (E34881g, A09449g). Expectedly, E19745g coding for GSH transferase/peroxidase, peptide-methionine (R)-S-oxide reductase (C07077g), and two oxidoreductases (C20251g and D02444g) were strongly upregulated upon exposure to uranium to diminish the consequences of oxidative stress. Likewise, upregulation of genes involved in DNA damage repair, like D11666g, E23386g, F22077g involved in mismatch repair, *RCC1* (E15583g) involved in chromatin condensation, and *RRN3* (D35575g) which is an RNA polymerase I-specific transcription initiation factor. As observed macroscopically in the previous study (Kolhe et al. 2020), exposure to uranium was accompanied by structural changes. Transcriptome profiling revealed two genes putatively involved in that process: 1,3-β-glucanosyltransferase (A03597g) involved in the elongation of 1,3-β-glucans chains, and chitin synthase (B16324g). Severe stress imposed in that study caused cell cycle arrest, which was hallmarked by downregulation of cytoskeletal-binding protein *HOF1* (E29557g) that maintains the mitotic actinomyosin contractile ring at the plasma membrane, chromosome segregation gene *KIN3* (E34067g) having serine/threonine kinase activity, but also subunits of condensine: *SMC2* (F24783g), *SMC4* (C19129g), *YCS4* (F06402g), subunit 2 (B03476g), and *YCG1* (F08679g). Decreased expression of these genes (and several others involved in cell cycle regulation) is known to arrest cell division in the G2 phase and prevent G2 to M-phase transition.

Park and Nicaud (2019) provided evidence for the implication of *RTS1* (E00154g) and *MFS1* (E03872g) genes in developing tolerance to a volatile FA – propionate (at 40 g L^−1^), in *Y. lipolytica*. Genomic library screens allowed to identify two novel genes, undescribed for *Y. lipolytica* in this context previously. The first one, E00154g is a homologue of *S. cerevisiae RTS1* regulatory subunit of protein phosphatase 2A (PP2A). *S. cerevisiae Δrts1* genotype exhibits sensitivity to temperature, ethanol, sorbate, and osmotic pressure. Overexpression of *RTS1* in *Y. lipolytica* conferred tolerance also to other weak organic acids such as lactate, formic acid, malic acid, and succinic acid. The second identified gene, E03872g, was similar (~ 48%) to MFSs, which facilitate the transport of a variety of substrates, including ions, sugar phosphates, drugs, amino acids, peptides, and toxic substances, across cytoplasmic or internal membranes. In addition, MFS transporters regulate and are regulated by the stress response machinery and control membrane potential and/or internal pH (discussed above). Three MFSs were identified in *Y. lipolytica*: E03872g, C08228g, and A15774g; but the other proteins bearing MFS-like domain were also described in this yeast (C01001g, D02014g, F19492g, C08965g, and D00407g; upregulated in Kolhe et al. (2021)). A strain overexpressing *MFS1* exhibited higher tolerance to propionate (withstanding even 50 g L-1) than the one overexpressing *RTS1* or the control. On the other hand, overexpression of *RTS1* provided the strain with higher tolerance to lactate, formic acid, malic acid, and succinic acid than the control strain, which was not observed for the *MFS1*-overexpressing strain.

An interesting global view on *Y. lipolytica*’s response to toxic chemicals was provided by Walker et al. (2019), who studied the yeast cell response to ionic liquids. The authors used an adaptive laboratory evolution approach to develop a strain able to grow in the presence of high concentrations (up to 18%) of 1-ethyl-3-methylimidazolium acetate ([EMIM][OAc]), which was a unique trait among different microbial species (Ryu et al. 2015). Under exposure to the ionic liquid, a wild-type cell developed cavities, dents, and wrinkles along the surface, visualized in SEM images, hallmarking severe damage to the cell envelope. In contrast, the evolved strain retained native, untreated morphology. Physiological characterization and omics analysis demonstrated that the biggest contribution to the developed phenotype was by a restructuring cell wall and its membrane. Specifically, it was discovered that sterols and conferred by them membrane’s rigidity played the key role for the exceptional performance of the evolved strain. Omics analyses revealed that sterol biosynthesis was the only “lipid pathway” significantly perturbed in the modified strain, in addition to amino acid biosynthesis/degradation. The unexposed, mutated strain had greater basal glycerophospholipid content, synthetized C16:1 FA, that was absent from the parental strain, and more C18:1, which was deemed to increase membrane fluidity. Upon exposure to the ionic liquid, ∼twofold increase in ergosterol content was observed for the resistant strain, which was not the case for the wild type. Analysis of transcription level of genes involved in the sterol biosynthesis pathway (*STER*1*/*A10076g; *STER*2*/*E15730g; *STER*3*/*F04378g; *STER*4*/*B05126g; *STER*5*/*B23298g; *STER*6*/*F11297g; *STER*7*/*C22165g; *STER*8*/*B17644g; *STER*9*/*F08701g; *STER*10-1*/*E32065g; *STER*10-2*/*B17204g; *STER*11*/*D20878g; *STER*12*/*A18062g; *STER*13*/*D19206g) showed that without exposure to the toxic compound, their expression was either not different from the control, or slightly downregulated. On the other hand, when the evolved strain was exposed to 8% [EMIM][OAc], 11 of the 14 sterol biogenesis pathway genes were significantly upregulated > twofold, with 7 of them (*STER5, STER6, STER8, STER10-1, STER10-2, STER11* and *STERTF* (B15818g)) upregulated > fourfold. Such massive and rapid upregulation was not observed in the wild-type strain, where only one gene *STER10*-1 was upregulated in the presence of the toxic compound.

### Osmo-active compounds

Molecular mechanisms of hyperosmotic stress response through the HOG (high-osmolarity-glycerol) pathway, and its direct association with filamentous and invasive growth (FIG) pathway, as well as mating/pheromones sensing/signaling pathway, in yeast are well-described. For details, the reader is referred to one of the excellent review papers focused specifically on osmo-stress response in yeast (Hohmann 2002; Clotet and Posas 2007; Saito and Posas 2012; Tatebayashi et al. 2020). In brief, hyperosmolarity response is operated by MAPK kinases cascade. The central element, Hog1p kinase (names of *Y. lipolytica* homologues are given in parentheses: E25135g), executes both cytoplasmic and nuclear functions. In the nucleus, it initiates transcription of stress-response genes by direct phosphorylation of many transcription factors, which frequently serve as chromatin anchors for Hog1p, localizing it in direct proximity to DNA. Among known interaction partners of Hog1p kinase are transcription factors Smp1p, Sko1p, Msn2p/Msn4p (acting through STRE DNA motives of, e.g., *CTT1*), or Hot1p, which is a direct activator of expression of *GPD1*, *GPP2* involved in GLY synthesis, and *STL1* – a GLY importer. In addition, Hog1p is known to directly interact with Rpd3p HDAC (E22935g/E08822g) and Rsc1/2/3p, a member of the SWI/SNF chromatin-remodeling complex, which is recruited by Hog1p to specific osmotic-responsive genes. Upstream sensing and signaling of HOG1 follows a general scheme for multistep phosphorelay, which can be initiated via either Sln1p (C21340g/F08789g) or Sho1p (D04048g) osmosensor branch. Operation through Sln1-Ypd1(C04928g)-Ssk1/2 (MAPKKK; A05247g) drives cytoplasmic functions of Hog1 and hyper-osmolarity response, while Sln1-Ypd1-Skn7(D14520g) directs nuclear functions of Hog1p and response to hypo-osmotic conditions and oxidative stress response. Initiation via Sho1p is later passed by Ste11p kinase (MAPK; F13629g) to Pbs2p (B15906g) and finally to Hog1p, or depending on small adaptor kinases, like Cla4p (C22770g), Ste5p or Ste20p (F00572g), can phosphorylate Ste7p (B15906g) and finally Kss1 (kinase of FIG pathway) or Fus3p (mating/pheromone sensing; E23496g). Cytoplasmic functions of Hog1p comprise, for example, stabilization of stress-response transcripts. The kinase directly binds to coding regions of stress-response genes in a 3’-UTR region-dependent manner, which is essential for an increased association of RNA Pol II with the transcript. It was shown that the half-life of stress-response mRNA may be selectively extended depending on the phase of response initial shock, induction, or recovery. In this sense, the signaling kinase affects elongation and transcription beyond initiation. Finally, the extent and length of transcriptional/ proteomic stress-response are regulated by the Ubp3 ubiquitin protease (F00638g; also directly phosphorylated by Hog1p), which manages turnover of specific transcription factors and/or RNA Pol II. Hog1p itself is dephosphorylated by PP2C phosphatases Ptc1p and Ptp2 (F30943g/F24585g; acting solely in the nucleus). To secure energy, materials, and time required for adaptation to stress conditions, Hog1p induces a rapid and transient delay at various stages of the cell cycle to permit the full development of adaptive responses before cell cycle progression resumes. While commonly associated with hyperosmolarity, the HOG pathway is implicated in the cellular response to cold, low pH, arsenite, acetic acid inhibitors of GPI, and sphingolipid synthesis.

Molecular mechanisms described above have been studied to a great extent in *S. cerevisiae*. However, many of the homologues genes involved in hyperosmolarity-response have been identified in *Y. lipolytica* through genome sequencing, global transcriptomics, and proteomics (Yang et al. 2015; Pomraning et al. 2016, 2018), and functional studies. Response to hyperosmolality is also probably the best studied at molecular/physiological level stress-response in *Y. lipolytica*. The high interest is a consequence of wide industrial exploitation of *Y. ipolytica* in the production of the valued chemical compound, ERY, which is promoted under these conditions (Rymowicz et al. 2009; Groenewald et al. 2014; Fickers et al. 2020; Rakicka-Pustułka et al. 2020). Additionally, *Y. lipolytica* cultivations are frequently conducted under high substrate load, which is convenient from a technical point of view. Fed-batch cultivations, which are the preferred mode of operation for rs-Prots production, may intrinsically bear large amplitudes in the culture medium osmolality. Studies by Rzechonek et al. (2018) detailed specificities of Hog1p (E25135g) operation. By generation of *Δhog1* and *HOG1*-overexpression stains, comprehensive characterization was enabled. It was evidenced that *Δhog1* exhibits increased resistance to the cell wall damaging agents, most probably due to abolished interference between Ste11(F13629g)-Pbs2p(B15906g)-Hog1 and Ste11(F13629g)-Ste7(B15906g)-Kss1 signaling pathways; but the deletant was more sensitive to NaCl (0.2–0.9 M) or sorbitol (0.2–0.3 M). Moreover, *Δhog1* mutation resulted in faulty cytokinesis, which was observed in a medium supplemented with 0.9 M NaCl, and bility to grow in a presence of 1 M GLY on solidified medium. In contrast, the *HOG1* overexpression strain was more resistant to heat stress (35 ℃) and menadione-induced oxidative stress, but not to osmotic stress or H_2_O_2_-mediated oxidative stress, illustrating complexity and intertwining of stress response cascades. Some further interesting insight was provided by the following studies on *Δhog1* mutant (Rzechonek et al. 2020). The strain was screened for growth in the presence of 0.5 and 0.75 M GLY, GLUC, and ERY. Strikingly, it occurred that ERY provides the strain with osmoprotection and reverses growth limitation under hyperosmolarity to a native level. Neither proline nor MAN could substitute ERY in this. Thus, it was inferred that ERY is a mediator of a specific, yet not fully characterized, HOG-independent osmoprotection in *Y. lipolytica*. To get a deeper insight, the authors generated two strains: *∆eyd1* deleted in ERY dehydrogenase (F01650g), and *∆euf1*, deleted in transcription factor mediating expression of ERY synthesis genes (F01562g). Deletion of *EYD1* disabled growth in media with ERY as the sole carbon source. Neither of the deletions impacted sensitivity to osmotic stress. Combined genotype *∆euf1∆hog1*/*∆eyd1∆hog1* made the strains sensitive to high osmolality induced by 0.5–1.0 M GLY and 0.75–1.0 M GLUC; but interestingly, additional supplementation with ERY reversed the sensitivity in GLY-based medium for *∆eyd1∆hog1* and *∆hog1* strains. The former could use ERY only as compatible solute and not as carbon and energy source. While the details of this HOG-independent, ERY-driven osmoprotection mechanism are not yet explained, these studies definitely shed new light on the mechanisms of osmo-stress response in *Y. lipolytica*.

One substantial difficulty in the direct comparison of literature data on osmo-stress response in *Y. lipolytica* is that it may be induced by different agents, and that the awaken response significantly depends on it. Among these are GLY (most frequently used for induction of ERY synthesis, as it can serve as a carbon source and a direct “precursor” for ERY), NaCl (which is very efficient in high osmolality induction but simultaneously causes salt-stress), sucrose (which can be utilized by some *Y. lipolytica* bearing a dominant reporter *SUC2* gene), and sorbitol (which is the most inert, as cannot be consumed by *Y. lipolytica*). A direct comparison of *Y. lipolytica*’s response to these factors was studied (Kubiak et al. 2019). Depending on the type of the inducer, supplemented at the concentrations within the upper limit of a tolerated range, different osmolality (1–4 Osm kg^−1^) and biological responses were stimulated. For example, the osmolality of 2–2.5 Osm kg^−1^ induced by 5% NaCl and 250 g L^−1^ of sorbitol both limited growth of the cells, but the former also inhibited the synthesis of a reporter protein, while the latter promoted it. Correspondingly, significant growth limitation was observed under 200–250 g L^−1^ of GLY (3.5–4 Osmg kg^−1^), but it induced enhanced synthesis of the reporter protein by threefold. Therefore, it seems highly relevant to report both the osmolality level and the type of a chemical osmo-inducer.

Andreishcheva et al. (1999) studied cellular processes underlying salt adaptation in an osmo- and salt-tolerant *Y. lipolytica* strain (able to grow in the presence of 12% NaCl), isolated from leaves of the salt-excreting arid plant. High salt concentration (9% =  ~ 310 Osm kg^−1^) was associated with a significant decrease in cell size, which allowed for a rapid concentration of intracellular solutes. The intracellular concentration of amino acids was threefold higher in the cells exposed to 6 and 9% of NaCl; the most upregulated were proline and alanine. On the other hand, no substantial changes to the total level of lipids (triacylglycerols and sterols) or their unsaturation index were observed. More insight into the molecular landscape awakened in *Y. lipolytica* by hyper-osmolarity was provided by a total proteome analysis (Yang et al. 2015). The stress was stimulated by the addition of 3% NaCl to a high salt medium, so that the resultant hyperosmolality equaled 4.21 Osm kg^−1^. As observed, any increase in osmolality above 4.15 Osm kg^−1^ limited growth but, expectedly, promoted synthesis of ERY. The hyperosmolality condition resulted in 54 DAPs, corresponding to 44 identified proteins. The highest upregulation (> fivefold) was observed for erythrose reductase Gcy12p (B07117p), which belongs to aldo–keto reductases (AKRs) involved in pentose phosphate pathway, also assigned to respond to osmotic, oxidative, and heat stress. Upregulation of these biological processes was additionally supported by nearly threefold upregulation of transketolase Tkl1p (E06479p) and Gcy15p (F18590p; nearly twofold upregulation). Furthermore, upregulation of a panel of proteins involved in glycolysis (e.g., Tpi1p, F05214p, 2.97-fold increase), TCA (AcnAp/AAT92542, > threefold increase; Mdh2p/E14190p, > twofold increase) and respiration (Cox4p, E19723p 1.56-fold increase; Mcr1p, D11330p, 1.83-fold increase) illustrates enhanced energy requirement of the cells exposed to hyperosmolality stress (estimated to be 15–30% higher). On the contrary, downregulation of Idh1p (E05137p, twofold decrease) and Sdh1p (D11374p, 1.45-fold decrease), two NAD + -dependent enzymes from TCA, may suggest sequestration of reducing equivalents fluxes. Hyperosmolality promoted synthesis of typical oxidative-stress responsive proteins, like Ctt1p (E34749p and E34265p), Sod2p (B08921p), Alkyl hydroperoxide reductase (Ahp1p, E25091p), as well as a panel of heat shock response proteins: Sti1p (C08987p), Hsp20p (C03443p), Hsp12p (D20526p), and mitochondrial localized DnaJ Mdj1p (F12551p). On the other hand, ER-localized Hsp70 Grp78p (E13706p) was significantly downregulated (threefold decrease). Grp78p is an ER chaperone involving in Ca^2+^ binding, misfolded protein degradation, and controlling activation of a transmembrane ER stress sensor. Interestingly, the osmotic stress modulated amino acids metabolism, by upregulation of Met6p (E12683p) and Shmtp (E16346p), but significant downregulation of Gdh1p (F17820p), which is the central node of amino acids metabolism, linking it to TCA at aKGA level. Another consequence of exposure to hyperosmotic pressure was the arrest of growth and downregulation of anabolic processes, like protein synthesis, hallmarked by downregulation of Tef1p (C09141g; > tenfold decrease), ribosomal 60S proteins L2 and L4, as well as seryl-tRNA synthetase (F02629p). Upregulation of Prb1p vacuolar protease (B16500p) marked intensive turnover of proteins, either misfolded under stress, or required as building blocks for the synthesis of stress-response proteins, or for release of free amino acids serving as osmo-protective compounds. Downregulation of membrane potassium channel (A00847p) hallmarks active adjustment of ions equilibrium. Another downregulated protein was sphingolipid long-chain base-responsive protein Lsp1 (C11341p), which together with Pil1 (identified as osmo-stress responsive in *Y. lipolytica* (Kubiak-Szymendera et al. 2021b)) are known to act in close proximity of cell membrane, and to be involved in the regulation of cell growth, heat stress responses, endocytosis, wall synthesis and repair, as well as repolarization of the actin cytoskeleton in response to stresses. Further insight into the *Y. lipolytica* osmo-proteome was provided in a recent study by Kubiak-Szymendera et al. (2021b). The main aim of that study was to establish if hyperosmolality can improve the synthesis of rs-Prot, as previous scientific reports brought contradictory results (Fiedurek 1998; Oganesyan et al. 2007; Lazar et al. 2011; Kubiak et al. 2019). Osmotic stress was simulated by the addition of sorbitol at 3 Osm kg^−1^. Macroscopically, the osmotic stress led to growth inhibition and over a threefold reduction in citric acid (CA) synthesis. On the contrary, MAN synthesis was increased by > sevenfold. Such a high upregulation was not observed for ERY (only twofold higher). Its concentration was similar in the treated and control variant (12 g L^−1^) at 24 h after the treatment; then, the polyol was re-consumed in the control variant due to the carbon source exhaustion. In terms of the target rs-Prot synthesis, the osmotic shock did not bring any significant change over the control; although it could be expected considering the high increase in chaperoning and folding capacity inferred from increased expression of genes: *HSP20*/C03443g; *STI1*/C08987g; *FMO1*/D22616g; *SSA6/7*/E35046g/D08184g. The onset of osmotic stress response was hallmarked by overexpression of three genes involved in TRE synthesis (*TPS1*/E14685g; *TPS2*/D14476g; *TPS3*/E31086g) and the AKR *GCY12* (B07117g) – which was the main upregulated protein in a proteome described by Yang et al. (2015). Interestingly, at the transcriptional level, no evidence for *HOG1* (E25135g) upregulation was observed, but its downstream targets, *SKN7* (D14520g) and *SKO1* (C16863g), were significantly upregulated. Considering changes at the proteome level, hyperosmolality led to the deregulation of 112 proteins (63 downregulated and 49 upregulated). The upregulated DAPs set was significantly enriched in protein refolding hallmarked by cytosolic Ssa6p/E35046p, Ssa8p/D22352p, Hsp104p/E27962p, Hsp90p/B15840p, ER-localized E25696p, F00880p, and mitochondrial Hsp78p/F12463p, Isu1p/B04928p (co-)chaperones induced by the factor of 1.23 to 3.8. Consistently to Yang et al. (2015), oxidoreductases, incl. AKRs involved in polyols synthesis, were among the most upregulated DAPs (Gcy13p/A15906p, Gcy12p/B07117p, A19910p, F24937p, D08778p), reaching average upregulation of > fourfold. Based on the global analysis of multiple DAPs identified in that study, it was inferred that the upregulation of AKRs involved in polyols synthesis was accompanied by the downregulation of proteins involved in TCA and FA synthesis. The treatment stimulated oxidative stress response operated by GSH peroxidase/E02310p, glyoxalase/F00682p, and mobilization of stored glycogen executed by glycogen phosphorylase (F04169p). Enhanced demand for energy under stress was highlighted by > twofold upregulation of proteins involved in mitochondrial respiration (D08602p, F24409p, D09933p) and transcriptional activation of *TPI1* (F05214g). Interestingly, DAPs involved in cellular membrane invagination and endocytosis (Pil1p/Lsp1p; C11341p/D13442p), vesicle transportation (B14102p, F27379p), and the major vacuolar protease Prb1p (A06435p) were all upregulated, consistently with data presented by Yang et al. (2015). It was suggested that upregulation of Pil1p was associated with increased endocytosis and sequestration of membrane channels and transporters, which were then targeted for proteolysis in vacuoles (upregulation of Prb1p). In contrast, the slightly marked downregulation of protein synthesis-related processes, represented previously by four DAPs, Tef1p (C09141g), ribosomal 60S proteins L2 and L4, and seryl-tRNA synthetase (F02629p) (Yang et al. 2015), was highlighted by a massive reduction of multiple DAPs levels in the osmo-proteome by Kubiak-Szymendera et al. (2021b). Among these were amino acid-tRNA (aa-tRNA) synthetases specific to isoleucine (A00264p), lysine (F16291p), proline (E05027p), leucine (E24607p), asparagine (E05005p), glutamate (E28468p), and tryptophan (B08943p), proteins involved in ribosome biogenesis (E31625p, F12661p), and biosynthesis of amino acids (Aro10p/D06930p, Bat2p/F19910p, Pro3p/B14399p), including sulfur compounds (CysK-Met25p/D25168p, MetBp/C22088p, B14509p). Downregulation of aa-tRNA synthetases limits translation and renders the already transcribed tRNAs uncharged. Uncharged tRNAs are known potent regulators of both transcription and translation (Raina and Ibba 2014; Gomez and Ibba 2020). In addition, high downregulation of elongation factor gamma (D16467p, B12562p) suggested the awaking of a specific, Rck2p-driven pathway for concerted downregulation of protein synthesis (Teige et al. 2001). Either higher resolution of comparative mass spectrometry (Kubiak-Szymendera et al. 2021b) vs. two-dimensional electrophoresis followed by mass spectrometry of selected spots (Yang et al. 2015) enabled more detailed characterization of these processes, or using a strain that overexpressed rs-Prot as a biological object subjected in the former study enhanced response within these biological processes. By comparison of the two osmo-proteomes, it is possible to deduce the general mechanisms that *Y. lipolytica* uses to fight against osmotic stress.

## Internal stress: synthetically forced over-synthesis of proteins

Apart from exposure to environmental threats, the rs-Prot producer cell may suffer from intensified metabolic burden triggered by synthetically forced over-synthesis of the target proteins. The latter, if not balanced, constitutes a significant stress factor impeding the optimal performance of the yeast producer. It has been demonstrated that heavy induction of the target protein synthesis leads to underperformance of the host cell in terms of growth and synthesis of the desired product (Graf et al. 2009; Puxbaum et al. 2015). In addition, it was shown recently that metabolic load caused by over-synthesis of two complex rs-Prots in *Y. lipolytica* significantly increased demand for the substrate, even at a reduced growth rate (Gorczyca et al. 2022). Increasing evidence suggests that depending on the biochemical properties of the target polypeptide, different molecular processes may be bottlenecked (Gasser et al. 2007; Nocon et al. 2014; Celińska and Nicaud 2019; Gorczyca et al. 2022).

Direct comparison of *Y. lipolytica* strains maintained in steady-state over-synthetizing individually one of four different reporter proteins under two different promoters provided substantial insight into protein-dependent burdens (Korpys-Woźniak et al. 2020). The proteins differed in size (~ 27 to ~ 65 kDa) and type of post-translational modifications (lack – YFP, highly glycosylated – TlG, and highly disulfide-bonded – SoA). Depending on the expression platform and promoter strength, but foremost, depending on the type of the over-synthesized protein, both transcription and the amounts of the final product (translation and secretion), and even morphology, differed substantially. The biggest metabolic challenge was imposed by the largest protein with a high number of O- and N-glycosylation sites (TlG). In this case, overexpression of *TlG* from the 4UASpTEF promoter led to a significant increase in its expression level and the amount of active, secreted protein (vs. hp4d), which happened at the expense of biomass growth and a significant increase in the synthesis of stress-response molecule – MAN. Still, the overall amounts of the target protein (calculated as % of nitrogen based on activity to unify the measures) were very low vs. YFP or SoA. The highest amounts were received for a small, not modified post-translationally secretory protein scYFP, and the disulfide-bonded SoA; therefore, these over-producing strains were termed high synthesis and secretion (*HSS*). Further analyses of that set of strains through global transcriptomics revealed the molecular background underlying these macroscopic observations (Korpys-Woźniak and Celińska 2021). In general, targeting a protein for secretion leads to an over sevenfold higher number of DEGs. Over-synthesis of inYFP resulted in a very low number of DEGs (327), meaning that it did not constitute a substantial challenge for the cell, but the level of the target polypeptide (YFP) was ~ tenfold lower when compared to scYFP (2199 DEGs). On the other hand, a relatively low number of DEGs (897) was found upon over-synthesis of TlG, but here, it hallmarked cellular stress and onset of UPR (*HAC1*, B12716g upregulated by ~ 1.25-fold). DEGs profile for *inYFP* strain was mainly composed of upregulated genes involved in ion homeostasis and downregulation of ribosomes biogenesis. *TlG* strain profile was enriched in only several more overrepresented functional categories, but it was also hallmarked with an increased abundance of vacuolar sorting factors *ATG8* (E02662g), *ATG13* (F03432g), *VPS70* (B05258g), and *CUP5* (F24475g); and vacuolar proteases *PEP4* (F27071g), *PRC1* (A18810g), *PRB1* (B16500g). Interestingly, many of these genes were also upregulated in in YFP strain, which was the less efficient producer of YFP when compared to scYFP. It could be speculated that limited cellular capacity disallowed further accumulation of the target protein and its degradation. On the contrary, protein degradation, autophagy, and vacuolar protein sorting were all downregulated in *scYFP* and *SoA* strains (the HSSs). These strains were also characterized by enhanced expression of genes localized to mitochondria, including *MDJ1* (F12551g), *MGE1* (C18513g), *HSP60* (F02805g), and *HSP10* (B05610g), as well as by significant upregulation of oxidative stress response genes, including *CTT1* (E34749g), thioesterase (B14575g), *HSP42* (C03443g), and glyoxalases *HSP31* (F00682g, C22000g). Expression profile of these genes illustrate that overproduction of rs-Prots is a heavily energy- and material-consuming process, and additionally inherently associated with oxidative stress, as observed for many other yeast species, as well (Tyo et al. 2012; Delic et al. 2013; Hou et al. 2014; Martínez et al. 2016). Oxidative stress response was only initiated in the *HSS* strains. It is well recognized that overproduction of heterologous secretory protein frequently leads to endogenous oxidative stress. The major driver of ROS generation in the secretory pathway is the oxidative folding process, taking place in the ER lumen. Noteworthy, over-synthesis of proteins lacking disulfide bonds, so not subjected to oxidative folding, may also induce a massive oxidative stress response in *Y. lipolytica*, as evidenced in (Korpys-Woźniak and Celińska 2021). Co-overexpression of a transcription factor Hap1 involved in managing aerobic metabolism in yeast, targeting genes involved in oxidative stress response, relieved the ongoing stress and allowed to enhance the synthesis of a target heterologous protein (Martínez et al. 2016).

From the transcriptomics data presented by Korpys-Woźniak and Celińska (2021), it was clear that the highly producing cells enter a growth arrest phase (G1 phase) as a strategy undertaken by the cells to withstand the high burden imposed on them. This hypothesis was supported by significant upregulation of “negative regulation of cellular macromolecule biosynthesis” biological process in general, but also many other discrete changes, like downregulation of *CLN1* G1/S-specific cyclin (C15114g; promoting G1 to S transition) gene, activation of detoxification processes (common upregulation of peroxisomal channel PXMP2/4), or high upregulation of F22187g gene involved in homeostasis maintenance. On the other hand, the *HSS* strains were characterized by high downregulation of the *RSFA* (E26763g) gene encoding a ribosomal silencing factor, enabling adaptation to stationary phase conditions by downregulating protein synthesis, which is one of the most energy-consuming processes. Its downregulation released ribosome assembly from the inhibitory action of RsfAp. Intriguingly, *RSFA* was not significantly downregulated in TlG and inYFP, which were also overproducing r-Prot. Consequently, transcriptomes of *TlG*/*inYFP* strains were hallmarked by downregulation of ribosome biogenesis, rRNA processing, etc.

Based on a direct comparison of the effects of over-synthesis of two secretory proteins significantly differing in molecular weight (55 vs. 120 kDa) at transcription, translation, and secretion levels, Swietalski et al. (2020) were able to note several interesting observations. Namely, they found that the larger protein’s transcripts tended to accumulate, indicating insufficient translation capacity. While that was not the case for the smaller protein, here, the process was limited at the secretion level, as the polypeptide accumulated inside the cell in high quantities. Limited translation of the larger protein relived the secretory pathway load, and consequently, the majority of synthesized active protein could be secreted outside. Indication of these mechanisms and bottlenecks facilitates understanding of frequently observed lack of linearity in the transcription-translation-secretion pipeline.

Different processes within the secretory pathway were targeted for engineering with the aim to relieve the internal burden caused by the over-synthesis of heterologous secretory protein (Korpys-Woźniak et al. 2021). The choice of the target genes was guided by previous omics analysis (Korpys-Woźniak and Celińska 2021). Overexpression of the target scYFP was combined with genes involved in the synthesis and chaperoning of the nascent polypeptides (*RPL3* /C21560g, *SSA5* /F25289g, *SSA8* /D22352g), folding (*PDI1*/E03036g, *SLS1*/E32703g, *CNE1*/B13156g) or transportation (*YET3*/E26026g, *USO1*/D23947g, *SEC1*/E22044g, *SSO1*/E23243g, *CWP11*/E22286g), co-cloned individually. The major initiator of UPR (*HAC1*/B12716g) was also included in the experimental set. In addition, two thermal conditions were adopted, 25 ℃ and 30 ℃. Co-overexpression of the “secretory helpers” involved in translation (*RPL3*) and chaperoning activity (*SSA5* and *SSA8*) contributed to significantly increased intracellular accumulation of the reporter (inYFP), but its secretion was improved only under the decreased temperature. Corresponding results were obtained with syntaxin (*SSO1*), where the accumulation of the reporter was significantly higher at 30 ℃, but the protein’s secretion was more efficient under 25 ℃. Interestingly, the combined action of the *SSO1* overexpression and 30 ℃ resulted in the highest level of extracellular YFP under this temperature. It was thus inferred that fusion of the Golgi-derived vesicle to plasmalemma is the bottleneck of the secretory pathway under the regular temperature of *Y. lipolytica* cultivation, which can be alleviated by either *SSO1* overexpression or decreased temperature. On the other hand, co-expression of *USO1* (involved in vesicle-mediated ER to Golgi transport, which is most frequently pointed to as a key bottleneck in the secretory pathway) brought no (25 ℃) or negative (30 ℃) effect of on the secreted amounts of YFP. Likewise, co-expression of the most straightforward targets, frequently adopted as secretion enhancers in fungi, namely transcription factor *HAC1* and ER-resident chaperones *PDI1*, *SLS1*, and *CNE1*, resulted in controversial outcomes. Upon co-expression of *HAC1*, *SLS1*, and *CNE1,* any improvement in the expression level of the *YFP* gene could be seen under 30 ℃, but it was not associated with an increase in protein synthesis and secretion. It was very surprising, as co-overexpression of *HAC1* is the most frequently employed strategy for relieving the internal stress caused by overexpression of secretory proteins. It was demonstrated that overexpression of *HAC1* improves the secretion of heterologous reporter proteins in *S. cerevisiae* (Duan et al. 2019) and *P. pastoris* (Guerfal et al. 2010). Co-overexpression of another UPR stress-relieving factor, *PDI1*, brought temperature-dependent effects, and moderate improvement (~ 30%) in YFP secretion could be seen only after temperature downshift. It suggested that the decreased temperature slowed down transcription which gives sufficient capacity to the translation and folding machinery to correctly process the nascent polypeptides. Altogether, co-expression of genes involved in the synthesis of polypeptides relieved the metabolic load and enhanced levels of the target protein in a temperature-independent manner; for efficient secretion of the protein retained inside the cell in large amounts, the secretory pathway had to be released by temperature downshift. Co-expression of the genes involved in the protein trafficking did not significantly improve the amounts of the target polypeptide. Nevertheless, their overexpression allows to maintain the secretory pathway’s capacity under not favorable thermal conditions (*SSO1*, *CWP11*).

An interesting approach to relieving internal stress encountered by co-overexpression of three secretory proteins has been recently proposed by Wei et al. (2019, 2021). Considering that ER is the central organelle for all protein synthesis, lipid biosynthesis, and lipid droplet formation, the authors pointed out that enforced overexpression of secreted proteins will cause a drain on the ER, leading to competition among synthesis/secretion of the protein and lipid synthesis (which was the applicatory aim of that study). In addition, it was noted that overexpression of three secretory proteins was associated with the accumulation of saturated FAs, which is a marker of ER stress. Correspondingly, significant changes in the lipid metabolism due to high level over-synthesis of the abovementioned scYFP and SoA proteins were observed (Korpys-Woźniak and Celińska 2021). The genes involved in lipid metabolism were found to be downregulated, corroborating the concept by Wei et al. (2019) on competition within the ER lumen. The initial approach aiming at relieving the internal stress by Wei et al. (2019) was to modify the medium composition by increasing the C/N ratio. Limited nitrogen provision decreased protein synthesis and relieved ER stress, but enhanced synthesis of the target lipids. Furthermore, the authors supplemented the high C/N ratio medium with a chemical chaperon (trimethylamine N-oxide dihydride), which facilitated more fluent protein folding and lowered the ER and oxidative stresses. In the following study (Wei et al. 2021), the sucrose non-fermenting 1 (*SNF1*) gene was deleted to release lipid and protein biosynthesis processes from its repressing activity. Based on the observed lower accumulation of saturated FAs and improved extracellular activity of the three target proteins, it was concluded that the internal stress was relieved by generating the *ΔSnf1* genotype.

## Summary

Synthesis of heterologous proteins as well as resistance to stress factors are both biological traits of high relevance to biotechnological production exploiting *Y. lipolytica*. Based on the molecular landscape of the awakened responses, it can be concluded that these two biological processes remain strongly interconnected and subjected to tight cross-talk. Specificities of cellular response to a particular stressor, like “heavy metal,” “increased temperature,” or “over-synthesis of disulfide-bonded protein”, are detailed above, to the extent that was enabled by the literature data. Though it seems that the systematic division into specific stress factors and responses employed here may be artificial, as the biology and the key players of the stimulated stress responses frequently remain the same. Hence, in summary, brief general characteristics of a “stressed *Yarrowia*” cell will be given below and are illustrated in Fig. 1.

Primarily, as shown in the experiments with elevated temperature, oxidative and toxic chemical agents, and dehydration, *Y. lipolytica* is more prone to “get stressed” when grown in exponential phase rather than stationary. This characteristic is related to the thicker cell wall, higher content of sterols, and increased length of lipid chains in the cell membrane of the stationary-phase cells. In addition, the chromatin structure of cells growing in the log phase is more relaxed and exposed to damaging agents. Some of the ultrastructural changes developed as a stress response in *Y. lipolytica* cells are corresponding to physiological phenomena occurring upon the transition of the cells to the stationary growth phase, without exposure to a stress factor. Furthermore, the reviewed literature data provide characteristics of a stressed phenotype. As frequently observed, following exposure to a stress factor, the cells encounter a temporary growth arrest, over which the response/adaptation is developed. In addition, under exposure to osmotic stress, translation is abolished, but heat stress led to an increased abundance of Tef1p – a marker of ongoing translation. It was frequently highlighted that yeast-to-mycelium transition is related to a defense mechanism, as it provides a selective advantage over ovoid morphotype, when facing conditions of stress. The onset of the dimorphic transition is associated with unipolar growth, asymmetric division, large, polarly located vacuoles and repression of cell separation after division. In the specific case of osmostress, the cells are shrinking to induce immediate concentration of cytosol, which is associated with rapid action of cell membrane pumps and cytoskeleton. Indeed, the majority of omics data highlight significant enrichment of cell wall biogenesis, polarized growth, and cytoskeleton (dis)assembly within the deregulated biological processes, irrespective of the type of stressor. At the ultrastructural level, stressed cells are characterized by enlarged mitochondria, enhanced number and volume of peroxisomes, and formation of lipid and polyphosphate granules in the cytosol. From the reviewed literature, it also clearly stems that synthesis/accumulation of MAN, TRE (and aKGA to a lesser extent) is a general strategy for combating the stress, irrespective of its type. MAN and TRE seem to serve as uniform stress response molecules due to multiple functions they provide, including antioxidant activity, preventing oxidative damage by scavenging oxygen radicals, protein, and phospholipid stabilizer, as they are known to mechanically interact and stabilize proteins and membranes, but also energy and carbon reserve, which can be assimilated when the growth recommence. Likewise, enhanced abundance/activity of ROS scavenging enzymes (Ctt1p, Sod2p, GAPDH, or Glr1p) is a general stress response strategy identified in *Y. lipolytica* exposed to different types of threats. Finally, it was observed that in response to different stressors, cell membranes alter their lipid composition by increasing the relative content of monounsaturated FAs, but the accumulation of saturated FAs is a marker of ER stress. The degree of unsaturation of acyl residues in phospholipids determines the fluidity of the membrane lipid bilayer, which in turn may influence the yeast’s survival and adaptation to environmental threats.

## Data Availability

For the purpose of Open Access, the author has applied a CC-BY public copyright license to any Author Accepted Manuscript (AAM) version arising from this submission.
